# Clinical Diagnosis of Red Cell Membrane Disorders: Comparison of Osmotic Gradient Ektacytometry and Eosin Maleimide (EMA) Fluorescence Test for Red Cell Band 3 (AE1, SLC4A1) Content for Clinical Diagnosis

**DOI:** 10.3389/fphys.2020.00636

**Published:** 2020-06-19

**Authors:** Ahmar Urooj Zaidi, Steven Buck, Manisha Gadgeel, Miguel Herrera-Martinez, Araathi Mohan, Kenya Johnson, Shruti Bagla, Robert M. Johnson, Yaddanapudi Ravindranath

**Affiliations:** ^1^Children’s Hospital of Michigan, Detroit, MI, United States; ^2^Wayne State University School of Medicine, Detroit, MI, United States

**Keywords:** red blood cell, anemia, membrane, Hematology, erythrocyte

## Abstract

The measurement of band 3 (AE1, SLC4A1, CD233) content of red cells by eosin-5- maleimide (EMA) staining is swiftly replacing conventional osmotic fragility (OF) test as a tool for laboratory confirmation of hereditary spherocytosis across the globe. Our group has systematically evaluated the EMA test as a method to screen for a variety of anemias in the last 10 years, and compared these results to those obtained with the osmotic gradient ektacytometry (osmoscans) which we have used over three decades. Our overall experience allowed us to characterize the distinctive patterns with the two tests in several congenital erythrocyte membrane disorders, such as hereditary spherocytosis (HS), hereditary elliptocytosis (HE), Southeast Asian Ovalocytosis (SAO), hereditary pyropoikilocytosis (HPP) variants, erythrocyte volume disorders, various red cell enzymopathies, and hemoglobinopathies. A crucial difference between the two methodologies is that osmoscans measure red blood cell deformability of the entire sample of RBCs, while the EMA test examines the band 3 content of individual RBCs. EMA content is influenced by cell size as smaller red cells have lower amount of total membrane than larger cells. The SAO mutation alters the EMA binding site resulting in a lower EMA MCF even as the band 3 content itself is unchanged. Thus, EMA scan results should be interpreted with caution and both the histograms and dot plots should be analyzed in the context of the clinical picture and morphology.

## Introduction

Our ability to accurately recognize the mechanistic basis of red cell disorders is continuing to evolve, none more so than the erythrocyte membrane disorders and enzymopathies. The human erythrocyte is the most abundant cell in the human body (Bianconi et al., 2013) and perhaps the most studied cell. There are approximately 20 major proteins and over 800 minor proteins in the red blood cell membrane. Integral membrane proteins are organized around band 3, an anion-exchange channel. The membrane skeleton, primarily composed of spectrin, actin and its associated proteins complete the composition of the phospholipid bilayer enabling it to maintain its shape (Lux, 2016). There are several other proteins that manage the regulation of volume and hydration that are implicated in rare, but important, disorders of the red blood cell membrane (Andolfo et al., 2016).

**FIGURE 1:**
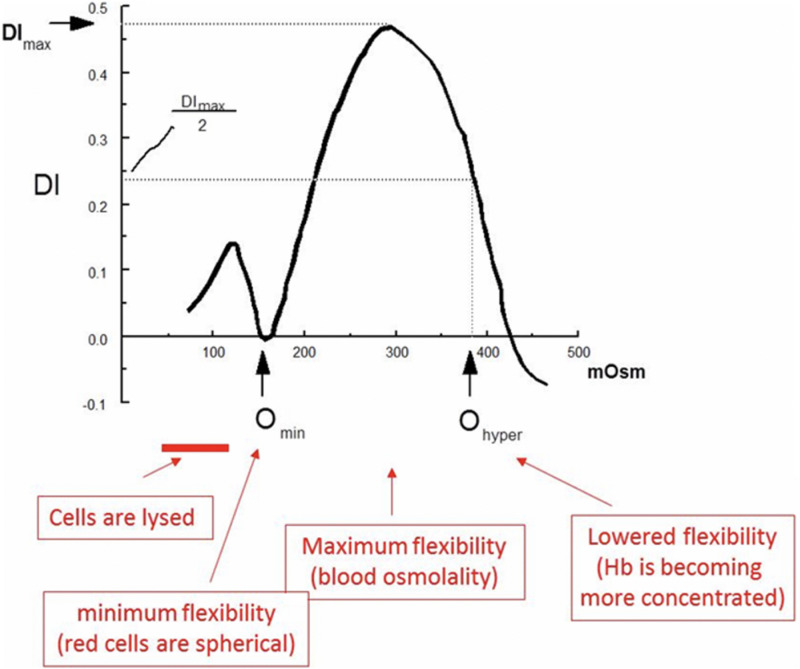
Several key features of the osmoscan allow us to understand the deformability properties of the red blood cells being tested. The most significant measures, are the O_min_, DI_max_ (*y*-axis the value of the index at isotonicity (or the deformability index maximum, DI_max_) and O_hyper_. The O_min_ is the point at which red blood cells have attained their critical hemolytic value, due to osmotic shifting of water into the cell in a hypotonic environment; beyond this point, the now spherocytic red blood cells would lyse with a further decrease in osmolality adapted with permission from Johnson and Ravindranath (1996).

There have been many important contributions to red blood cell membrane science since the original osmotic fragility (OF) test (Hunter, 1940). One such contribution came almost four decades ago, the ektacytometer (Bessis et al., 1980). The ektacytometer is a laser diffractometer that measures the deformability potential of a population of red blood cells over an osmotic gradient, and allows the characterization of many of the common red blood cell membranopathies. Red cells undergo shape change from discoid to elliptocyte configuration as they traverse through the capillaries and the micropores in the splenic sinusoids. This *in vivo* phenomenon is mimicked in the ektacytometer. The cells are exposed to an increasing osmotic gradient, and cell deformability (shift from discoid to elliptical shape) is gauged by how light scatters as the cell responds to shear forces. The result of this test, is a characteristic graph (the Osmoscan), that shows the amount of deformability on the *y*-axis, and osmolality on the *x*-axis (Figure 1; Mohandas et al., 1980).

The major components of the red cells that determine deformability are the biconcave shape of the red cells, the membrane fluidity and the internal viscosity of the cell. There are several key features of the osmoscan that allow us to understand the deformability properties of the red blood cells being tested (Clark et al., 1983). The most significant measures, are the O_min_, DI_max_ (*y* axis the value of the index at isotonicity or the ellipticity/deformability index maximum, EI/DI_max_), and O_hyper_. The O_min_ is the point at which red blood cells have attained their critical hemolytic value, due to osmotic shifting of water into the cell in a hypotonic environment; beyond this point, the now spherocytic red blood cells would lyse with a further decrease in osmolality. Thus, the EI at O_min_ measures the changes in surface to volume (S/V) ratio. The deformability index maximum is the index value at isotonicity, and the point at which red blood cells have attained the maximum ellipticity (DI_max_, EI_max_). Deformability index reflects the membrane integrity and elasticity. The O_hyper_ is the osmolality at which the index is midway between the maximal deformability and O_min_. O_hyper_ is increased in states of cellular hydration either because of decreased mean corpuscular hemoglobin concentration or net increase of water content (such as stomatocytosis/cryohydrocytosis). These values, when compared to normal red blood cells provide unique signatures in cells with membrane pathologies. However, despite the ability of the ektacytometer to aid in the clinical diagnosis of red blood cell membrane disorders (Groner et al., 1980; Johnson and Ravindranath, 1996), its use was limited until recently because of the non-availability of the original Technicon made instruments. A newer clinical grade version with digitized osmoscans, LoRRca Maxsis^®^, has become available and its usage will expand. Even with the new-generation ektacytometer gaining increasing popularity, the ability of centers to perform this test remains limited and yet approved by US FDA for clinical testing (Da Costa et al., 2016).

Almost 20 years after the introduction of ektacytometry, King et al. (2000) devised a simple alternative to standard OF test for laboratory confirmation of HS by using eosin maleimide fluorescence to measure band 3 content (AE1, SLC4A1). HS cells lose membrane through vesiculation, and along with it band-3 protein. The test uses the functional property of the fluorochrome, eosin-5-maleimide (EMA), which covalently binds to the 430th residue (lysine) on the first extracellular loop of band 3 (Cobb and Beth, 1990). This test provides investigators with insight on the amount of band 3, or functional loss of membrane, that exists in pathologic red blood cell. The results of the EMA test are reported as mean channel fluorescence (MCF) compared to a control sample- MCF ratio (patient’s MCF/control MCF). However, as described below, the slope of the EMA curve on both sides indicates valuable information on the variation in cell size; trailing shoulders on the right side is prominent in patients with high reticulocyte count and the leading shoulder on the left indicates presence of fragmented red cells. Thus, EMA test should be interpreted not solely on the MCF, but also on the overall pattern of EMA intensity. The measurement of band 3 content by flow cytometry has enabled for rapid diagnosis of spherocytosis in intact red blood cells and is fast replacing the OF test.

A crucial difference between the ektacytometry and EMA test is that osmoscans measure red blood cell deformability of the entire sample of RBCs, while the EMA test examines the band 3 content of individual RBCs. One is a measure of deformability under shear stress (function) and the other an estimate of a critical membrane protein (structure). Further, the ektacytometer is a test that simulates flow of red blood cells through the vasculature, and is able to capture the spirit of the dynamic red blood cell; the test is a measure of RBC geometry, cytoplasmic viscosity, cell volume regulation and fluidity of the membrane (Mohandas et al., 1980). The testing is limited by the availability of the instrument, the need for specialized staff and the need to delay analysis after transfusion (Da Costa et al., 2016). The EMA is user-friendly, with quick turnaround time of testing, and flow cytometers are readily available in most institutions. Though the EMA only describes reduction in band 3 protein and does not interact with other integral proteins, it can serve as a reliable indirect measure of cytoskeletal health (King et al., 2004) but mild defects may result in indeterminate results (Bolton-Maggs et al., 2012).

Our group has systematically evaluated both the EMA test and osmoscans obtained as a method to screen for a variety of anemias over the past 10 years and compared these results. Our experience allowed us to characterize distinctive patterns in a variety of congenital hemolytic anemias (red cell membrane disorders, erythrocyte volume disorders, enzymopathies/hemoglobinopathies) and as well in some acquired disorders. We have not systematically evaluated the OF test by flow cytometry (Won and Suh, 2009; Crisp et al., 2012). In this review, we will describe both the ektacytometry-osmoscans and EMA tests from patients and the lessons we have learned from practical experience.

## Materials and Methods

All individuals studied were patients at the Children’s Hospital of Michigan/Wayne State University School of Medicine, and the data has been collected from routine clinical testing. This review was approved by the Wayne State University Human Investigation Committee. We reviewed records of children with suspected red blood cell membranopathies or anemias who underwent both osmotic gradient ektacytometry and flow cytometry studies using eosin-5-maleimide from 2007 to 2017. In all cases, the peripheral blood smears were reviewed by members of the division of Pediatric Hematology/Oncology. Osmotic gradient ektacytometry was performed on an ektacytometer manufactured by Technicon Instruments (Miles Diagnostics, Tarrytown, NY, United States). This instrument includes two pumps to generate buffer gradients, a microprocessor that controls the viscometer motor and gradient pumps, an image analyzer and a keyboard with a display. For standard clinical testing, the rotor speed was set at 150 RPM, operating at a shear stress of 159.3 dynes/cm^3^ (all of the tests were done by Gerard Goyette until 2015 and following his untimely death by MG). The analog curves were digitized (done by MHM and KJ) and O_min_ and O_max_ values were used to compare diagnostic reliability in dominant HS and general characteristics of the curves relative to normal were used to describe changes in other membrane disorders. For EMA flow cytometry, cells were stained in the dark for 30 min at room temperature with agitation, washed with 1 ml cold PBS, and re-suspended in 0.5 ml PBS plus fixative (PBS plus 0.5% formaldehyde). Acquisition was performed on a Coulter XL Flow Cytometer (Coulter Corp., Miami, FL, United States) equipped with a 488 nm Argon laser. Results were analyzed using EXPO-32 software (by SB and MG). We reviewed complete blood counts on the days of the sampling and entered them into a database without patient identifiers.

### Cases

Table 1. Number of patients for each disorder.

**TABLE 1:** Number of cases.

43	Hereditary Spherocytosis
8	Hereditary Elliptocytosis
3	Hereditary Pyropoikilocytosis
3	ABO Incompatibility
4	AIHA
3	Southeast Asian Ovalocytosis
3	Erythrocyte Volume Disorders

### Hereditary Spherocytosis (HS)

**FIGURE 2:**
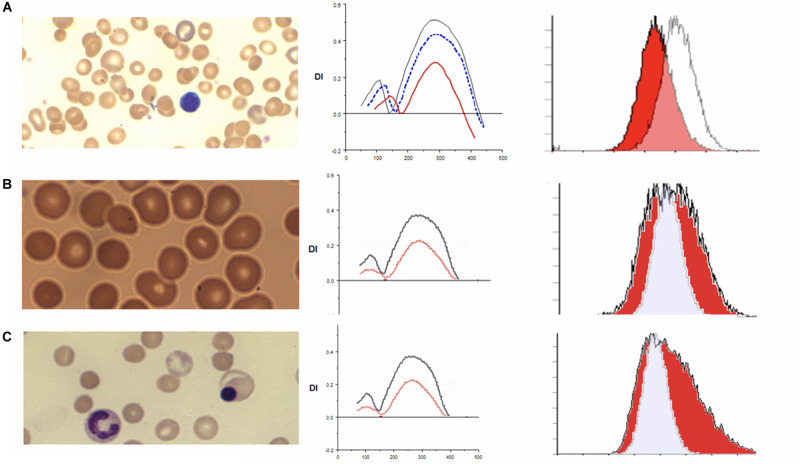
**(A)** Depiction of smear in left panel, Osmoscan (blue line: mild HS, red line: severe HS) in middle panel, and EMA histogram in right panel in cases of hereditary spherocytosis. **(B)** Depiction of smear in left panel, Osmoscan in middle panel, EMA histogram in right panel in cases of ABO Hemolytic Anemia. **(C)** Depiction of smear in left panel, Osmoscan in middle panel, and EMA histogram in right panel in cases of autoimmune hemolytic anemia.

Hereditary spherocytosis (HS) is the most commonly inherited red blood cell membranopathy with significant clinical heterogeneity. Features like anemia, jaundice and splenomegaly are common, and jaundice may be the only sign in neonates (Ribeiro et al., 2000; Delaunay, 2007; An and Mohandas, 2008; Nussenzveig et al., 2014; Andolfo et al., 2016; Da Costa et al., 2016). Hydrops fetalis is an exceedingly rare complication in HS (Gallagher et al., 1995; Ribeiro et al., 2000). While the molecular defects are protean, in general, it is the weakened vertical linkages between the membrane skeleton and the lipid bilayer’s integral proteins that drive this disease process (Palek and Lux, 1983; Perrotta et al., 2008). This disruption of linkage between the lipid bilayer and the cytoskeleton via ankyrin (band 2.1) results in echinocyte formation. More specifically defects in ankyrin, band 3, beta spectrin, alpha spectrin or protein 4.2 cause spherocytosis through loss of membrane surface area. As erythrocytes age they begin to exhibit a variety of membrane abnormalities including the loss of potassium and water, leading to cell dehydration and increased cell density, and the loss of surface area, likely owing to gradual release of membrane microvesicles (Lux, 2014). The spherocyte generates characteristic results on osmoscans with the entire curve being inside of the control-O_min_ is increased, EI/DI_Max_ is lower and O_hyper_ is reduced. The three aspects of HS cells driving these changes are the loss of surface area, reduced S/V ratio and the high internal viscosity (Clark et al., 1983). The loss of band 3 content results in decreased EMA binding. The MCF (mean channel of fluorescence) is reduced variably with the histogram shifted to left (see Figure 2).

In 43 patients with HS, the EMA MCF was 395.69 ± 53.48, whereas in 76 normal controls the value is 514.50 ± 15.25 (Zaidi et al., 2015; Table 2). Thus compared to controls HS is easily diagnosable disease by either EMA or osmoscan. The severity of spherocytic defect, can be depicted in an osmoscan based on the degree of reduction in the y-coordinate of the elongation index’s maximum point (Johnson and Ravindranath, 1996). Figure 2A shows a typical osmoscan of three samples: control (black line), mild HS (blue line), and severe HS (red line). In patients with low MCF and this typical osmoscan tracing, a diagnosis of HS is certain. There is good correlation between values for O_min_, S/V ratio and MCF in dominant HS (Zaidi et al., 2015). However, in our cohort of patients, we were unable to find any replicable association or relationship with clinical indicators of severity- hemoglobin/hematocrit, reticulocyte counts, bilirubin and other red blood cell parameters (MCHC, MCH, MCV, RDW) with either O_min_ or MCF values. This suggests that phagocytosis of damaged red cells and the extent of compensatory erythropoeitic response are critical additive determinants of clinical severity. Molecular testing was done only in a few cases and thus we cannot make sweeping comments, but the reader is referred to a recent publication by the Dutch group (Huisjes et al., 2019). Lowest MCF values in HS cases were seen in a teenager with band 3 mutation (SLC4A1; NM_000342.3; c.2423G > T, pArg808Leu) and in a child with ankyrin mutation (ANK1 NM-000037.3; c.2023dup;pVal675Glyfs^∗^118). Arginine mutations appear to cause severe band 3 deficiency because of decreased incorporation of band 3 protein in to the lipid bilayer (Palek and Lux, 1983; Gallagher, 2013). However, the teenager (status post splenectomy) with SLC4A1 Arg808Leu had a hemoglobin of 17.8 g/dL, MCF 301.1 (ratio 59% when compared to mean control values) when tested at age 15.5 years. A young girl with ANK1 mutation had hemoglobin 6.5 g/dL at age 6 weeks and 8.5 g/dL at age 12 months with no interventions. Her brother had a hemoglobin of 10.3 g/dL at 18 months, and their father at age 26 had a hemoglobin of 13.6 g/dL; neither were transfused nor had splenectomy, and had the same mutation as the index patient. The EI_Max_ on the osmoscan was reduced to an equal degree in all three patients (51, 55, 56% of control).

**TABLE 2:** Hereditary spherocytosis.

HS (*n* = 43)			Normal controls (n = 76)		
	
	Mean	SD		Mean	SD
MCF	395.69	53.48	MCF	514.50	15.25
O_min_ (*X*-axis)	166.11	14.65	O_min_ (*X*-axis)	147.67	6.90
DI_MAX_ (*Y*-axis)	0.34	0.08	DI_MAX_ (*Y*-axis)	0.49	0.05

Neither the osmoscans nor the EMA histograms can distinguish dominant HS from recessive HS. In the only child with recessive HS we have evaluated, the EMA MCF was 348 with MCF ratio of 0.87; the child had two alpha spectrin mutations (SPTA1 c.3267A > T, p.Y1089X and alpha-LEPRA [c.4339-99C > T]), in trans; the case was included in two recent publications on recessive HS (Chonat et al., 2015; Gallagher et al., 2019).

Heterozygotes with EPB42 mutations exhibit milder changes from our limited experience (2 cases) consistent with published data (Kalfa et al., 1993). Thus, in general, our findings are in agreement with the detailed structure-function correlations reported by the von Wijk laboratory (Huisjes et al., 2019).

### Spherocytes in Immune (Allo/Auto) Hemolytic Anemias

Immune hemolytic anemia is the result of antibody mediated destruction of red cells. It can be caused by maternally transferred alloantibodies as in neonates with Rh sensitization and A/B blood group infants born to type O mothers. In older children and adults acquired autoantibodies can cause hemolytic anemia. Variable numbers of spherocytes are present on smears. The osmoscan by itself cannot distinguish acquired from congenital spherocytosis, as is evident from the patterns shown in Figure 2. In ABO hemolytic disease, on the ektacytometer, the O_min_ and Di_max_ (*Y* axis) may appear similar to normal adult controls; neonatal red cells typically have high osmoscans (Johnson et al., 1999) and age matched neonatal controls usually are not available concurrently and thus a “normal” scan may actually indicate underlying spherocytosis (congenital or caused by antibody due to blood group incompatibility). An additional confounding variable is the high reticulocyte count. Evaluation of the slope of the curve on EMA test is frequently informative with leftward leaning curve indicating a gradual loss of membrane due to the antibody as opposed to the rapid loss caused by structural defects in HS where the whole curve is shifted left. This is consistent with the reported data on this topic (Johnson et al., 1999). Diagnosis of neonatal HS and distinction from spherocytosis associated with ABO incompatibility remains a challenge; family history is helpful and repeat testing 8–10 weeks postnatally should clarify the diagnosis in *de novo* congenital HS cases (see Figures 2B,C and Tables 3, 4).

**TABLE 3:** ABO hemolytic disease.

ABO (*n* = 3)			Normal controls (*n* = 76)		
	
	Mean	SD		Mean	SD
MCF	554.40	42.97	MCF	514.50	15.25
O_min_ (*x*-axis)	175.39	25.34	O_min_ (*x*-axis)	147.67	6.90
DI_MAX_ (*y*-axis)	0.31	0.02	DI_MAX_ (*y*-axis)	0.49	0.05

**TABLE 4:** Autoimmune hemolytic anemia.

AIHA (*n* = 4)			Normal controls (*n* = 76)		
	
	Mean	SD		Mean	SD
MCF	497.63	66.84	MCF	514.50	15.25
O_min_ (*x*-axis)	169.24	6.53	O_min_ (*x*-axis)	147.67	6.90
DI_MAX_ (*y*-axis)	0.35	0.06	DI_MAX_ (*y*-axis)	0.49	0.05

### Hereditary Elliptocytosis (HE) and Hereditary Pyropoikilocytosis (HPP)

**FIGURE 3:**
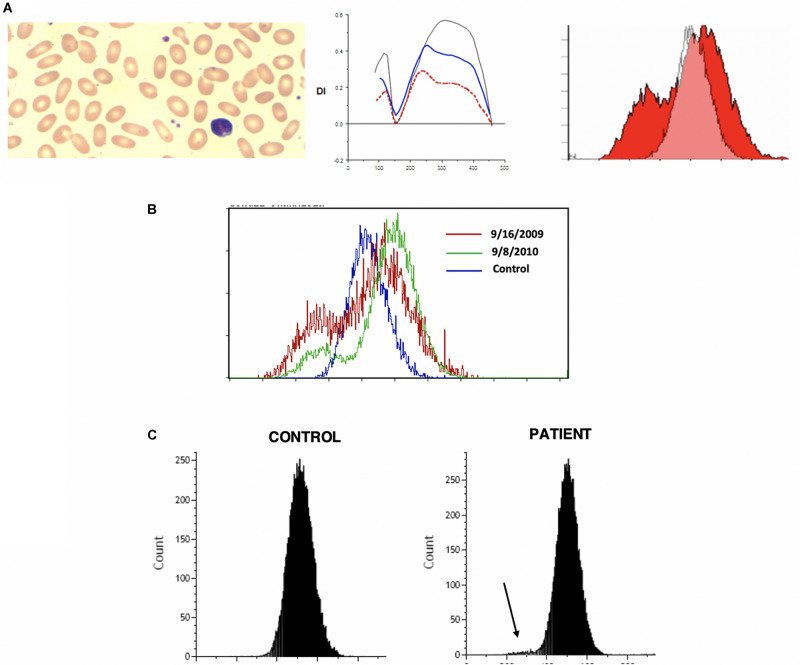
**(A)** Depiction of smear in left panel, Osmoscan in middle panel (blue line depicts a mild case, red line depicts a severe case), and EMA histogram in right panel in cases of hereditary elliptocytosis with severe fragmentation. **(B)** Changes in the EMA histogram in a HE patient with severe fragmentation at birth, and improvement 1 year later. **(C)** EMA in an older child showing a prominent left shoulder, but mostly resolved fragmentation.

Hereditary elliptocytosis (HE) is clinically heterogeneous disorder. The presence of elliptically shaped red cells on the peripheral blood smear is the characteristic feature of HE. HE patients can have a diverse spectrum of clinical findings ranging from life threatening anemias to asymptomatic carrier state (Lazarova et al., 2017). The inheritance of HE is autosomal dominant, with rare reports of recessive mutations. Homozygous and compound heterozygous HE variants present with moderate anemia and hemolysis (Delaunay, 2007; An and Mohandas, 2008). Weakened lateral linkages in membrane skeletons due to either defective spectrin dimer-dimer interaction or weakened spectrin-actin-protein 4.1R junctional complex results in the decrease of mechanical stability in these patients (Gallagher, 2004). Significant fragmentation of red blood cells in addition to the classic elliptocytes are a feature of the disease (see Figure 3). Newborn infants may show high level of red cell fragmentation, so called hemolytic HE (as seen in Figure 3A), which improves by age 1–2 years (as seen in Figure 3B; Palek and Lux, 1983; Mentzer et al., 1987; An and Mohandas, 2008). In the family with 4.1 deficiency, homozygotes had more severe disease than heterozygotes.

Hereditary elliptocytosis cases show distinctive indented plateau on osmoscans pattern (Tchernia et al., 1981; Johnson and Ravindranath, 1996; Silveira et al., 1997). HE cells generate a trapezoidal profile in an osmoscan, with a normally positioned O_min_ and O_hyper_ but diminished EI_Max_. The truncated curve reflects the inability of the already elliptical cells to deform further under shear stress. These findings were conformed in more recent work (Suemori et al., 2015) and has been re-appraised with the use of the new generation ektacytometers (Da Costa et al., 2016).

**FIGURE 4:**
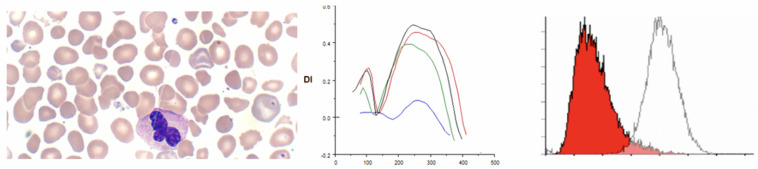
Depiction of smear in, Osmoscan (blue line: HPP, black line: control, red/green line: father/mother), EMA histogram in cases of hereditary pyropoikilocytosis.

On EMA scans a bimodal pattern comprised of distinct population of cells with decreased MCF (representing fragmented cells) and a population with normal to high MCF may be present, especially in neonates. The EMA histograms reflect the level of fragmentation seen on smears. In infants who show high numbers of fragmented cells (“hemolytic HE”) two distinct populations one with low MCF and another with high MCF) can be identified (Figure 3A). Often in such cases the level of fragmentation decreases as the infant gets older and the EMA histograms reflect this with a decrease in the size of the red cell population with low MCF (Figure 3B). This feature also distinguishes hemolytic HE cases from cases of HPP where the extreme fragmentation gives a single population of cells with low MCF (Figure 4). In older children and adults the curve may overlap the control or shifted slightly to right but a trailing population of fragment cells can identified on the left shoulder of the curve (Figure 3C). This is an important finding as, it expands the value of the EMA test beyond the diagnosis of HS. In a recently reported work, heterogeneity in the ovalization of the HE patients had no association with EMA binding (Suemori et al., 2015).

**FIGURE 5:**
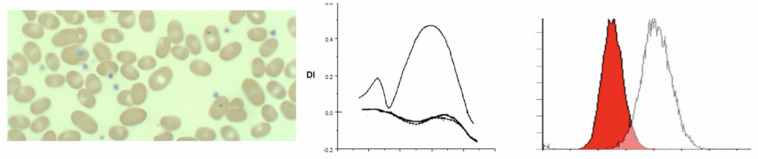
Depiction of smear in left panel, Osmoscan in center panel, and EMA histogram in right panel in cases of Southeast Asian ovalocytosis.

Hereditary pyropoikilocytosis (HPP), originally thought to be a unique and separate disease process, has been reclassified as a subset of HE due to double heterozygosity of mutations in the alpha-spectrin gene (Gallagher, 2004). This severe subset of HE-type disorders, in which red blood cells appear like those seen in thermal burn patients (Zarkowsky et al., 1975), is characterized by neonatal jaundice and transfusion dependent hemolytic anemia that persists through life. A peripheral smear shows microspherocytosis or micropoikilocytes more than elliptocytes. A protein analysis of the phospholipid bilayer of HPP reveals a mild spectrin reduction but a greater increase in spectrin dimer content than in common HE (Zarkowsky et al., 1975). We have seen 3 families with HPP phenotype. In one family despite the name of the disorder we were unable to show heat instability by morphology or circular dichroism studies in purified spectrin (Ravindranath and Johnson, 1985). Molecular studies (courtesy of Bernard Forget, Yale University) showed double heterozygosity for mutations near the dimer-dimer association site in alpha spectrin (Alpha1 74 c.28 CGT to CAT p.Arg > His and Alpha1 50a at c209 CTG > CCG, Leu to Pro). There was significant reduction in alpha spectrin. In these patients (*n* = 3) there was extreme cellular fragmentation and transfusion requirements abated only after splenectomy. We noted the lowest MCF values, with MCF 213.9 ± 52 in these cases, consistent with the MCV values of <50 fl. There is also a very clear change in the O_min_ and the deformability index was severely decreased (see Figure 5). The red cells in HPP appear to have a significantly decreased ability to maintain deformability in the face of hypotonicity, evidenced by their critical hemolytic value occurring at an osmolality much higher than normal controls and HS cases. These parameters distinguish HPP cases from common hemolytic HE especially in infants where the fragmentation could be mistaken for HPP. Both osmoscans and EMA test separate the two entities (see Tables 5, 6).

**TABLE 5:** Hereditary elliptocytosis.

Hereditary elliptocytosis (*n* = 8)			Normal controls (n = 76)		
	
	Mean	SD		Mean	SD
MCF	478.69	28.42	MCF	514.50	15.25
O_min_ (*x*-axis)	147.59	21.06	O_min_ (*x*-axis)	147.67	6.90
DI_MAX_ (*y*-axis)	0.22	0.05	DI_MAX_ (*y*-axis)	0.49	0.05

**TABLE 6:** Hereditary pyropoikilocytosis.

Hereditary pyropoikilocytosis (*n* = 3)			Normal controls (*n* = 76)		
	
	Mean	SD		Mean	SD
MCF	213.85	52.26	MCF	514.50	15.25
O_min_ (*x*-axis)	175.75	13.44	O_min_ (*x*-axis)	147.67	6.90
DI_MAX_ (*y*-axis)	0.11	0.14	DI_MAX_ (*y*-axis)	0.49	0.05

### Southeast Asian Ovalocytosis (SAO)

The first report of Southeast Asian Ovalocytosis (SAO) was over five decades ago (Eng, 1965) in malaria endemic regions of Papua New Guinea and Laos. It has now been described in other South East Asian populations and in African Americans (Ravindranath et al., 1994). The condition is inherited in a dominant fashion and genetic studies generally reveal heterozygotes. Subsequently, the genetic lesion has been found to be related to a mutation in band 3 (anion exchanger 1; SLC4A1) (Tse and Lux, 1999). The mutation results in the deletion of eight amino acid residues in band 3, that result in misfolding of the first transmembrane domain (Tanner et al., 1991). Most patients present with minimal hemolysis, though neonatal hyperbilirubinemia is described. Patients will have stomatocytic elliptocytes, that are pathognomonic to SAO (Liu et al., 1990; Tanner et al., 1991; Cheung et al., 2005). Ektacytometry studies show completely non-deformable red blood cells, with no discernable deformability across a wide osmotic gradient, resulting in a characteristic near flat curve (Mohandas et al., 1984; Ravindranath et al., 1994). EMA testing presents a unique signature as the 8 amino acid deletion in the first *trans* membrane loop of AE1 renders lysine 430 inaccessible to binding with EMA, band 3 content itself is not decreased (Moriyama et al., 1992). The reduction of MCF in our three patients (one Philippino, two African Americans) with SAO from normal controls was approximately 35% of control MCF value, and approximately 16% lower from HS patients. This indicates the EMA can be used as a screening method for SAO, although ektacytometry is more specific (see Figure 5 and Table 7).

**TABLE 7:** Southeast Asian ovalocytosis.

Southeast Asian ovalocytosis (*n* = 3)			Normal controls (n = 76)		
	
	Mean	SD		Mean	SD
MCF	334.13	22.01	MCF	514.50	15.25
O_min_ (*x*-axis)	NA	NA	O_min_ (*x*-axis)	147.67	6.90
DI_MAX_ (*y*-axis)	−7.33	6.43	DI_MAX_ (*y*-axis)	0.49	0.05

### Erythrocyte Volume Disorders

**FIGURE 6:**
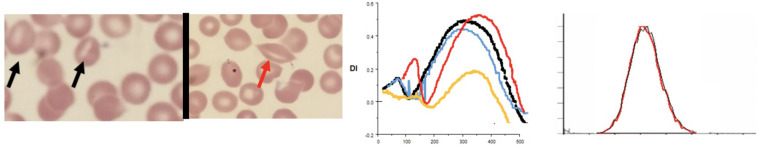
Depiction of smear (black arrows point to classical stomatocytes and red arrow to a “lipstick” stomatocyte, Osmoscans in middle panel- black line: control; yellow line: hereditary spherocytosis, red line: overhydrated stomatocytosis [PIEZO1 (p.Arg1728Cys)], and blue line: dehydrated stomatocytosis [PIEZO1 (p.Thr1589Ile)], EMA histograms in right panel in cases of stomatocytosis overlap control histograms.

Erythrocyte volume disorders include hereditary xerocytosis (now referred to as dehydrated stomatocytosis) and stomatocytosis (overhydrated stomatocytosis) (Mohandas and Gallagher, 2008; Andolfo et al., 2016). A recent major discovery is the linkage of xerocytosis cases with mutation in the mechanosensitive calcium transporter PIEZO1 (Zarychanski et al., 2012). Other genes implicated in EVDs are the Gardos channel KCNN4 and potassium channel ABCB6. Anemia may be mild or in some cases mild erythrocytosis has been reported. The morphology is not always distinct with a variable mixture of xerocytes and stomatocytes. In three patients with erythrocyte volume disorders associated with PIEZO1 mutations we observed mixed patterns on osmoscans, similar to mild HS or a right shift of the curve suggesting increased cellular hydration (Knight et al., 2019). Classical xerocytosis with cellular dehydration results in a left shifted curve. In our experience the most common cause for a left shifted curve on osmoscans is in patients with hemoglobin C by itself or in combination with sickle hemoglobin (HbSC), consistent with the known effect of hemoglobin C on the Gardos channel (Hannemann et al., 2015). In our three patients with PIEZO1 mutations (c.4766 C > T p.Thr1589Ile, c.5182 C > T p.Arg1728Cys, c.6835C > T p.Arg2279Cys) EMA MCF scans were not diagnostic while Osmoscans were abnormal (Johnson and Ravindranath, 1996; Da Costa et al., 2013; Andolfo et al., 2016; Knight et al., 2019). The clinical findings of xerocytes (dense spherocytes) and stomatocytes coupled with left or right shifted osmoscan curves should raise the suspicion of erythrocyte volume disorders; mutation testing is necessary for confirmation. The variable Osmoscan pattern may reflect the diverse mutations in PIEZO1 and their impact on the calcium and cation fluxes (Knight et al., 2019; see Figure 6).

### Other Disorders With Low MCF Values on EMA Test

Red cells from individuals with iron deficiency anemia and thalassemias show low MCF values on EMA scan but can be distinguished from HS cases because of a left shoulder of smaller cells indicating the anisopoikilocytosis in these disorders. A left shoulder may also be seen in cases with hemolytic uremic syndrome and other thrombotic microangiopathies indicating the level of circulating fragmented red cells.

### RBC Enzyme Deficiency

**FIGURE 7:**
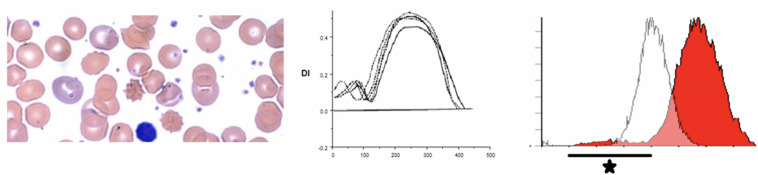
Depiction of a smear in left panel, Osmoscan in center panel, and EMA histogram in a case of pyruvate kinase deficiency.

In G6PD deficiency and glycolytic enzyme disorders ektacytometry shows high osmoscans indicating greater deformability of red cells (Johnson and Ravindranath, 1996). EMA test in patients with glycolytic enzyme deficiencies, in this case phosphoglycerate kinase 1, show higher MCF with right shifted curves reflecting the high MCV noted in these cases (low mean cell age) (Zaidi et al., 2019). In addition our review of patients with glycolytic enzyme deficiency (in 3 cases of pyruvate kinase (PK) deficiency (post splenectomy) and a new case of phosphoglycerate kinase (PGK1) deficiency) revealed a previously unsuspected signature -on scatter plots and histograms there is a distinctive tail of small cells – presumably the ATP depleted dense spiculated cells (Zaidi et al., 2019; see Figure 7).

## Conclusion

**FIGURE 8:**
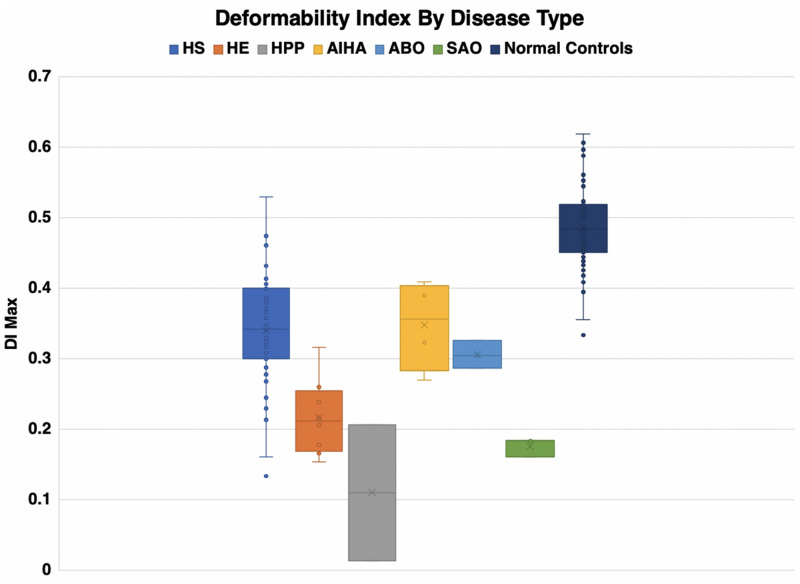
Deformability index by disease type.

**FIGURE 9:**
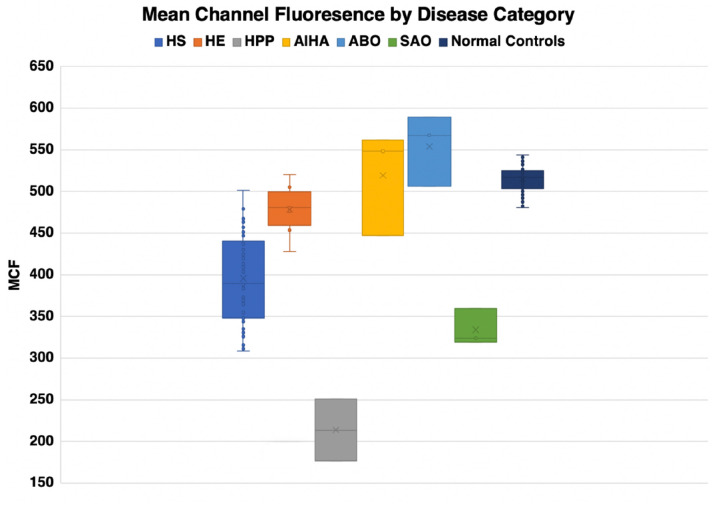
Mean channel fluorescence by disease type.

Eosin-5-maleimide testing and osmoscans are complementary and the combined information can lead to better diagnosis of red cell membrane disorders. There are very salient and key differences in the diagnostic ability of the EMA test and ektacytometry. The EMA provides a static, quantifiable measurement of the amount of band 3 protein, while the ektacytometer provides a fluid, physiologically simulated test that assesses red blood cell deformability in an active fashion (Figures 8, 9). In interpreting the EMA test, attention should be paid to not only the MCF value, but also the slope of the curve on either side, which together reflect the heterogeneity of cell size (Figure 2). The lowest MCF values were seen in HPP cases; SAO cases had MCF values intermediate between HPP and HS cases (Figure 9). In erythrocyte volume disorders EMA tests may be normal, but changes in cell hydration can be suspected better on the osmoscan. At present, the use of the Osmoscan is limited by the availability of the instrument, and the need for specialized staff. The EMA is user-friendly, with quick turnaround time of testing, and flow cytometers are readily available in most institutions. These tests can provide very different results, and should be used in combination with morphology on blood smears and blood counts including the red cell indices. Molecular testing is needed for confirmation of erythrocyte volume disorders.

## Data Availability Statement

All datasets generated for this study are included in the article/supplementary material.

## Ethics Statement

This review was approved by the Wayne State University Human Investigation Committee.

## Author Contributions

AM set up the EMA test. MH-M, SBa, and KJ digitized the osmoscans and determined the Omin, DImax, and O_hyper_ values. Ektacytometry testing was done under supervision of RJ. AZ wrote the manuscript. RJ gifted the ektacytometer to YR. YR supervises the red cell and flow cytometry laboratories. All authors contributed to the article and approved the submitted version.

## Conflict of Interest

The authors declare that the research was conducted in the absence of any commercial or financial relationships that could be construed as a potential conflict of interest.
